# Predictors of fear of COVID-19 in a low-income country: health literacy is important

**DOI:** 10.3389/fpsyg.2024.1394957

**Published:** 2024-07-31

**Authors:** Soheila Ranjbaran, Khalil Maleki Chollou, Salar Abedi-Nerbin, Towhid Babazadeh

**Affiliations:** ^1^Department of Public Health, Sarab Faculty of Medical Sciences, Sarab, Iran; ^2^Department of Nursing, Sarab Faculty of Medical Sciences, Sarab, Iran; ^3^Sport Management, Education Department of Ajabshir, Ajabshir, Iran

**Keywords:** COVID-19, fear, health literacy, low income, self-efficacy

## Abstract

**Introduction:**

Excessive fear of a threatening condition or situation can result in individuals engaging in unhealthy behaviors, especially during the COVID-19 pandemic. Identifying the influential factors of fear can be effective in designing and implementing health-center interventions to control and reduce the COVID-19 pandemic.

**Methods:**

This study was a cross-sectional design implemented in Iran in 2022. Participants were adults 18–65 years of age recruited randomly from primary Health Care Services Centers (HCSCs) by medical records (*n* = 380, 64.7% female, mean (SD) age 32.14 ± 10.98 years) in urban and rural areas of the county. The data collection included a demographic form, Fear of COVID-19 questionnaire, Perceived Barriers of COVID-19 preventive behaviors, Self-efficacy, and Health Literacy for Iranian Adults (HELIA) questionnaire.

**Results:**

It was determined that the fear of COVID-19 showed statistically significant differences according to gender (*p*-value = 0.013), education level (*p*-value = 0.001), and job status (*p*-value = 0.001). According to the hierarchical linear regression, educational level (*p*-value = 0.001; β = 0.665), job (β = 0.126; *p*-value = 0.018), self-efficacy (*p*-value = 0.015; β = 0.103), and Health Literacy (HL) (*p*-value = 0.001; β = 0.446) were significant predictors of fear of COVID-19. Also, among variables, educational level (*p*-value = 0.001; β = 0.665) and HL (*p*-value = 0.001; β = 0.446) were the most important predictors for fear of COVID-19.

**Discussion:**

This research shows that HL has a potential and determinative role in controlling fear of COVID-19. The findings can help healthy policymakers and healthcare providers design HL-based programs in similar epidemics and pandemic situations.

## Introduction

The World Health Organization (WHO), in 2020, declared the Chinese outbreak of COVID-19 to be a Public Health Emergency of International Concern, posing a high risk to countries with vulnerable health systems (Sohrabi et al., 2020). The large-scale deaths and adverse effects of the COVID-19 pandemic resulted in mental health problems, including stress and fear (Pourfridoni et al., 2022). WHO recommended enhancing whole-of-society coordination mechanisms to support preparedness and response to prevention and control strategies for the disease (WHO, 2020a,b). However, these recommendations and suggestions regarding controlling and reducing the COVID-19 pandemic led to more fear of diseases, perceived threats, and stress responses among people, and many healthcare providers and policymakers did not know the adequacy of the COVID-19 outbreak news (Shaukat et al., 2021; Pourhaji et al., 2022). Individuals were exposed to profound fear during the COVID-19 pandemic. Uncertainty about COVID-19 symptoms and incorrect or irrelevant information, the fear of being infected or of infecting others, rapid transmission, and changed routines and lifestyles have caused fear and panic in patients and others (Ahorsu et al., 2020; Buheji and Buhaid, 2020; Akbarpour et al., 2022; Ayaz-Alkaya and Dülger, 2022). The research results were conducted in Turkey, and the implied level of health literacy (HL) was a predictive factor of COVID-19 fear (Kazak and Ozkaraman, 2023). “HL represents the cognitive and social skills which determine the motivation and ability of individuals to gain access to, understand, and use the information in ways which promote and maintain good health”(Horowitz and Kleinman, 2008). In situations like the COVID-19 pandemic, HL has a central role in behavioral and emotional management (Abel and McQueen, 2020). The results of the research have indicated that Health Literacy (HL), e-Health Literacy (e-HL), knowledge, attitudes, reactions to stress, well-being and motivation are essential determinants of preventive health behavior (Glanz and Bishop, 2010; Fleary et al., 2018; Sánchez-Arenas et al., 2021; Ranjbaran et al., 2022; Rezakhani Moghaddam et al., 2022; Babazadeh et al., 2024), while low HL is associated with poorer health outcomes (Berkman et al., 2011) and more likely to have health risk behaviors (Wolf et al., 2007). An acceptable level of HL gives people a better understanding and interpretation of health conditions and challenges. Then, they can have better decision-making, reaction, and performance and look after themselves and those around them (Peyvand et al., 2020). Also, enough HL helps people achieve and understand the correct information regarding the pandemic, apply trustworthy health information, and reduce fear resulting from misinformation (Abdel-Latif, 2020; Shaukat et al., 2021). In fact, in contrast to biomedical vaccines, HL can apply as a social vaccine and public health strategy in order to prevent infection from COVID-19 and other similar diseases in the future (Okan et al., 2023). Individuals with accurate information and a high level of HL can better overcome their fear and adopt healthy behaviors. The study results in Turkey demonstrated COVID-19 fear related to HL (Türkoğlu et al., 2022). Fear and misleading information about COVID-19 diseases were challenging for people with insufficient HL worldwide (Ayaz-Alkaya and Dülger, 2022).

The psychological and behavioral responses of the general population play an essential role in the control of the outbreak (Qian et al., 2020). People who perceive greater risks are more willing to implement protective behaviors and more likely to prefer government policies designed to mitigate risk (de Bruin et al., 2020). Bandura explained that increased self-efficacy, perceived threat, perceived benefits, and decreased perceived barriers may increase the probability of health-related behaviors (Champion and Skinner, 2008; Thomas, 2011). Perceived barriers can directly lead to non-participation in preventive behavior (Rosenstock et al., 1988). A broad understanding of some key factors for understanding behaviors and behavior change can provide a foundation for well-informed public health programs, help identify the most influential factors for a particular person or population, and enable program developers to focus on the most salient issue (Shirzadi et al., 2016). It will be difficult to enhance HL during the epidemic and pandemic because of the immediate action, fear, and panic of governments, policymakers, and healthcare providers. Identifying the determinants of COVID-19 fear can help inform public health strategies to reduce the fear of COVID-19 diseases. Because even though fear factor can play a deterrent role, in many cases, it has an inverted effect in controlling diseases, especially horrible diseases. Fear of COVID-19 affects health-related quality of life (HRQoL) and perceived social support, and it is the most critical determinant of both physical and mental HRQoL (Kontodimopoulos et al., 2022; Yenen and Carkit, 2023).

Considering the fear role in the pandemic and the importance of HL, this study has focused on both fear and HL, emphasizing the critical role of HL. To control Coronavirus or other epidemics and pandemic situations in the future, it is necessary to comprehensively understand the factors associated with fear among people and present the correct healthy information by improving HL. Hence, this research aimed to investigate the predictors of fear of COVID-19 among adults and the important role of HL.

## Materials and methods

### Study design and participants

The present cross-sectional study was conducted in Sarab, a mountainous county in East Azerbaijan, the northwestern part of Iran. The population under study was the people 18–65 years of age covered by four governmental primary Health Care Services Centers (HCSCs) in urban and rural areas of the county. District Health System (DHS) provides Primary Health Care (PHC) to defined populations in Iran. The ultimate mission of DHS is the promotion of community well-being. Based on the National Health Care System (NHCS) in Iran, healthcare centers are divided into urban and rural healthcare centers. Every HCSCs delivers primary health care services (such as health education, maternal and child health, health nutrition education, and so on) to these populations (Mohammadi and Mohammadi, 2012). Multistage cluster sampling was employed to recruit 380 people from HCSCs. In the first stage of sampling, the 4 HCCs were considered as clusters, and then, in the second stage, the respondents were randomly selected from 4 HCCs. 380 people 18–65 years of age were selected via Morgan’s table and Cochran’s formula (Krejcie and Morgan, 1970) with an *N* = 40,000, d = 0.05, *p* = q = 0.5 and Z = 1.96. Individuals were randomly selected from each HCSCs based on the population’s health records.


$$n=\frac{Nz^{2}pq}{Nd^{2}+z^{2}pq}=380$$

The criteria for inclusion in the study encompassed individuals aged 18 to 65 who provided consent to participate, exhibited no psychological issues, and possessed the capability to respond to questionnaires. The exclusion criteria comprised incomplete questionnaires, disinterest in study participation, a background of psychiatric hospitalization, or referral to a mental health professional as a result of mental ailments.

### Measures

Data was collected from November 11, 2022 to February 28, 2023 through face-to-face interviews using a structured 5-sections questionnaire.

#### Socio-demographic characteristics questionnaire

This questionnaire included age, gender, marital status, educational status, job, and monthly income.

#### Fear of COVID-19 questionnaire

The Fear of COVID-19 Questionnaire (FCOV-19 Q) was used to assess the levels of fear of COVID-19 among the participants. FCOV-19 Q is a 5-item questionnaire developed by Veisi et al. (2020). This tool is a valid and reliable instrument that was also validated used among Iranian people (α = 0.81). The questionnaire was evaluated using a 5-point Likert scale ranging from 1 = “strongly disagree” to 5 = “strongly agree.” The total score is calculated by adding all items to the five items, ranging from 5 to 25, wherein higher scores indicate greater fear of COVID-19. For the current study, the estimate of internal consistency, as measured by Cronbach’s Alpha, was 0.79.

#### Self-efficacy to COVID-19 preventive behaviors

A validated tool with six items was used to measure self-efficacy to COVID-19 preventive behaviors (Salavati et al., 2021). This scale included a person’s confidence in adhering to COVID-19 protective behaviors (“social distancing, wearing a mask and …”), a five-point Likert-type scaling, ranging from one (strongly disagree) to five (strongly agree), was adopted. In the current study, Cronbach’s Alpha was obtained 0.74.

#### Perceived barriers to COVID-19 preventive behaviors

The barriers of the COVID-19 preventive behaviors scale was developed by Salavati et al. (2021) and consisted of 4 items. For the scale of perceived barriers, the items were rated on a five-point Likert-type scale ranging from 1 to 5 (1 = totally disagree through 5 = totally agree). The higher the score, the lower barriers to performing COVID-19 preventive behaviors were concluded. In this study, the Cronbach alpha value was 0.70.

#### Health literacy for Iranian adults

The validated Health Literacy for Iranian Adults (HELIA) was used in this research (Montazeri et al., 2014). This questionnaire consisted of 47 items and five dimensions: (1) Reading health information (4 items) measured using a five-interval Likert scale, ranging from 1 (completely difficult) to 5 (completely easy). The total score ranged from 4 to 20. The higher scores represented a high level of reading health information. (2) Understanding health information (7 items) rated on a 5-point scale ranging from 1 (completely difficult) to 5 (completely easy). The scores for understanding items ranged from 7 to 35. The higher scores determined the better condition for understanding. (3) Appraisal of health information (4 items), rated on a 5-point scale ranging from 1 (never) to 5 (always). The total scores for this dimension ranged from 4 to 20. The high scores indicated a better ability to appraise health information. (4) Ability to access health information (6 items) was scored on a five-interval Likert scale (always = 5, most of the time = 4, sometimes = 3, seldom = 2 and never = 1). The scores ranged from 6 to 30, and a higher score showed better access to health information. (5) Decision-making (12 items) was measured with a five-interval Likert scale (always = 5, most of the time = 4, sometimes = 3, seldom = 2, and never =1). The scores for decision-making items ranged from 12 to 60. The higher scores were recorded to show a better condition. Cronbach’s alpha for all of the dimensions of the questionnaire was measured >0.7 (0.72–0.89) (Montazeri et al., 2014).

### Ethics approval and consent to participate

This study received ethical approval from the Sarab Faculty of Medical Sciences (ethic code: IR.SARAB.REC.1401.001) and conformed to the Declaration of Helsinki ethics guidelines. All participants signed a written informed consent form following explanations of the study’s goals and methodology. At any stage during the study, participants were free to leave.

### Data analysis

We performed all the analyses using SPSS 16 (SPSS Inc., Chicago, IL, United States) and presented the data by mean (SD) and frequency (percent) for quantitative and qualitative variables, respectively. Before analyzing the data, the Kolmogorov–Smirnov test was used to determine the normality of the data. The data distribution was normal according to the significance level of more than 0.05 of the Kolmogorov–Smirnov test. Therefore, the quantitative variables were analyzed using bivariate analyses (i.e., the independent samples *T*-test and ANOVA). Pearson’s correlation coefficient was used to measure the relationship among self-efficacy to COVID-19 preventive behaviors, perceived barriers to COVID-19 preventive behaviors, HL, and fear of COVID-19. We also used the Kolmogorov–Smirnov test to test the normality.

Hierarchical linear regression analysis three-step was conducted to determine which variables can predict fear of COVID-19. In Step 1, the covariates (i.e., age, gender, education level, marital status, income status, and job) were entered into the models. Self-efficacy and perceived barriers were involved in the second step, along with the demographic variables. In the third step, we entered the demographic variables, Self-efficacy, and perceived barriers with HL in the analysis*. p* < 0.05 was considered significant.

## Results

Table 1 displays the demographics of the participants. The average age of the participants was 32.14 years (SD = 10.98), with the majority of them being under the age of 30 (55.3%), around 65 percent of the participants being female, and the majority of them having an academic degree. The majority of the samples were married couples. Also, in Table 1, it was examined whether the levels of fear of COVID-19 of participants showed a significant difference according to age, gender, educational status, married status, monthly income, and job. It was observed that women had higher fear of COVID-19 levels. It was found that employed and highly educated individuals were more fear of COVID-19 (*p*-value < 0.05).

**Table 1:** The relationship between demographical variables with fear of COVID-19 (*n* = 380).

Variables		*N* (%)	Me ± SD	*p*-value
Age	Lower than 30	210 (55.3)	20.71 ± 3.29	0.597**
	30 to 39	130 (34.2)	20.42 ± 3.29	
	40 and higher	40 (10.5)	20.28 ± 3.07	
Gender	Male	134 (35.3)	20.01 ± 3.74	0.013*
	Female	246 (64.7)	20.86 ± 3.10	
Level of education	Under diploma	115 (30.3)	19.53 ± 3.73	0.001*
	Diploma	101 (26.6)	20.21 ± 3.20	
	College	164 (43.2)	21.50 ± 2.64	
Marriage status	Married	262 (68.9)	20.79 ± 3.18	0.354**
	Single	118 (31.1)	20.45 ± 3.29	
Income (month)	Less than 200 dollars	298 (78.4)	20.42 ± 3.31	0.119
	200 dollars and higher	82 (21.6)	21.06 ± 3.01	
Job	Employed	264	20.94 ± 3.09	0.001*
	Unemployed	116	19.69 ± 3.47	

* One-way ANOVA; ** Independent sample *t*-test.

The bivariate associations for self-efficacy, perceived barriers, and HL with fear of COVID-19 are shown in Table 2. Using the Pearson correlation coefficient test, we discovered that fear of COVID-19 statistically had significant relationships with all variables except perceived barriers (*p*-value >0.05).

**Table 2:** Pearson correlation coefficient of self-efficacy, perceived barriers, and health literacy with fear of COVID-19 (*n* = 380).

Variables	1	2	3	4	Me ± SD	Possible range
1 = Self-efficacy	1				23.86 ± 3.70	12–30
2 = Perceived barriers	−0.024	1			14.87 ± 3.08	7–20
3 = HL	0.061	0.145	1		121.28 ± 25.83	74–249
4 = Fear of COVID-19	0.125*	0.058	0.549*	1	20.56 ± 3.26	5–25

*Correlation is significant at the 0.01 level (two-tailed).

The effects of demographic variables, self-efficacy, perceived barriers, and HL on fear of COVID-19 were investigated using a hierarchical linear regression analysis (Table 3). In step 1, demographic features explained 10.6 percent of the variation in fear of COVID-19 (*F* = 7.396; *p*-value <0.05). This step shows that fear of COVID-19 was significantly associated with education (β = 0.264; *p*-value = 0.001) and job (β = 0.126; *p*-value = 0.018). An additional 1.8 percent of the variation in fear of COVID-19 was explained by self-efficacy and perceived barriers as predictor variables (step 2) (*F* = 3.70; *p*-value >0.05). Step 3 included the addition of HL, which explained an extra 23.6 percent of the variance (*F* = 136.22; *p*-value >0.05).

**Table 3:** Hierarchical linear regression for prediction fear of COVID-19 through demographic characteristics, self-efficacy, perceived barriers, and HL (*n* = 380).

Variables	β	R2	R2 change	F change	SE	*p*-value	Collinearity Statistics	
							Tolerance	VIF
**Step 1**								
Age	0.005	0.106	0.106	7.396	0.146	0.931	0.789	1.267
Gender	0.103				0.366	0.055	0.822	1.216
Education level	0.264				0.198	0.001*	0.919	1.088
Marital status	0.016				0.373	0.760	0.786	1.272
Income status	0.043				0.396	0.391	0.951	1.052
Job	0.126				0.377	0.018*	0.845	1.183
**Step 2**								
Age	0.003	0.124	0.018	3.706	0.146	0.953	0.779	1.284
Gender	0.091				0.365	0.088	0.821	1.218
Education level	0.252				0.197	0.001*	0.896	1.116
Marital status	0.023				0.371	0.662	0.776	1.288
Income status	0.060				0.397	0.233	0.918	1.090
Job	0.129				0.374	0.015*	0.823	1.215
Self-efficacy	0.100				0.052	0.043*	0.920	1.086
Perceived barriers	0.082				0.007	0.098	0.972	1.029
**Step 3**								
Age	0.020	0.360	0.236	136.222	0.056	0.656	0.763	1.310
Gender	0.085				0.580	0.064	0.820	1.219
Education level	0.173				0.665	0.001*	0.891	1.123
Marital status	0.025				0.173	00.589	0.774	1.292
Income status	0.001				0.006	0.986	0.918	1.090
Job	0.045				0.320	0.324	0.823	1.215
Self-efficacy	0.103				0.109	0.015*	0.918	1.090
Perceived barriers	0.060				0.009	0.155	0.966	1.035
HL	0.560				0.446	0.001*	0.963	1.039

*p < 0.05.

## Discussion

Determining the influential factors related to fear is important in preventing diseases, especially infectious diseases, during the pandemic. So, this study was applied to investigate the predictors of fear of COVID-19 among adults and the important role of HL.

In this research, among the demographic characteristics, being female, education level, and job were determinants of fear of COVID-19. These findings confirm the results of Kontodimopoulos (2022) in Greek (Kontodimopoulos et al., 2022). Gender was recognized as one of the demographic characteristics affecting fear of COVID-19. This was consistent with the results shown by Patelarou et al. in five European countries (Greece, Albania, Cyprus, Spain, and Kosovo) (Patelarou et al., 2022) and in Turkey (Ayaz-Alkaya and Dülger, 2022). In general, in preventing the disease, women perform better than men (Lau et al., 2010). Also, higher education level led to reducing fear of COVID-19 among people in this study. According to this finding, better understanding and reducing fear of COVID-19 requires education level and health literacy. In people with a high level of education, more accurate knowledge about spreading a virus (Türkoğlu et al., 2022) and the perception that behaviors will effectively reduce the risk of infection (Qian et al., 2020). In Iran, COVID-19 preventive behaviors were more significant in urban areas than in rural ones (Shahnazi et al., 2020). Employed people are more likely to fear COVID-19. It can be because employed people are more exposed to COVID-19 in the workplace than unemployed people. For instance, nurses were more fearful of COVID-19 during the pandemic (Patelarou et al., 2022). Therefore, among the demographic factors, gender, education level, and job situation should be addressed in educational programs. In terms of the appropriateness of educational interventions, the designed content in educational programs for an illiterate and unemployed person should be different compared to a highly educated and employed person. To explain further, a complexly designed infographic about the fear of the COVID-19 pandemic will be almost unusable for a person with a low level of education.

According to the results of this research, fear of COVID-19 showed statistically significant relationships with self-efficacy and HL. These results were consistent with the other study in Turkey that reported an association between health literacy and fear of COVID-19 among older adults (Ayaz-Alkaya and Dülger, 2022). It means that people’s health literacy can impact their fear of COVID-19. Based on the study results in Iran, COVID-19 media literacy and usage of social media apps were determinants of COVID-19 fear (Barati et al., 2022). In terms of the determinant of HL in COVID-19 fear in this research, also, this result was comparable with previous studies conducted in Iran, Pourfridoni et al. (2022), conducted in Vietnam, Nguyen et al. (2020) among medical students and conducted in Taiwan, among school principals. (Duong et al., 2022). The COVID-19 outbreak has emphasized increasing health literacy to prevent the spread of infection because health literacy is important for preventing communicable diseases (Paakkari and Okan, 2020). Results of a study in Australia showed that lower health literacy in individuals led to misinformation about COVID-19 and vaccinations than people with appropriate health literacy (McCaffery et al., 2020). Reducing COVID-19 fear is a crucial factor to address in Iran. Because, fear of COVID-19 is a significant factor in determining resilience and future anxiety (Yıldırım et al., 2023). A low understanding of COVID-19 symptoms, less recognition of COVID-19 protective behaviors, and more problems in research information and understanding policymaker messages about COVID-19 disease result from lower health literacy in people (McCaffery et al., 2020).

Self-efficacy was the other predictor of COVID-19 fear. People with high self-efficacy can better overcome their fears and care for their own against COVID-19 disease. This result was supported by the findings of the previous studies that reported fear of COVID-19 had a negative impact on self-efficacy (Doanh et al., 2021; Okan, 2021; Yenen and Carkit, 2023). The finding of Bidzan et al. revealed low levels of self-efficacy related to a stronger tendency to experience COVID-19 anxiety (Bidzan et al., 2020). Self-efficacy is the situation-specific confidence that people can cope with high-risk situations without relapsing to their former behaviors (Glanz et al., 2008). Individuals should have an acceptable level of self-efficacy to overcome barriers to behavior (Glanz et al., 2008). Self-efficacy is a modifiable factor that influences protective behavior, and the effects of fear are minor among those who be efficacious, creating a pathway to fearless compliance (Jørgensen et al., 2021; Mo et al., 2021). Then, it is necessary to address this factor during an outbreak of the diseases in the future.

Despite the spread of rumors and misconceptions during the COVID-19 pandemic, health literacy, self-efficacy, and appropriate health behaviors by health centers and national media are necessary. It is normal that people have some fear of COVID-19 disease, but it should modify to a positive factor by the HL and promote self-efficacy in performing protective behaviors such as wearing a mask, keeping a social distance, staying home, not touching their face, etc.

In this study, the results of the hierarchical regression model indicated that demographic features and demographic variable accounts for about 10.6 percent of the variation in fear of COVID. This step shows that fear of COVID-19 was significantly associated with education and jobs. An additional 1.8 percent of the variation in fear of COVID-19 was explained by self-efficacy and perceived barriers as predictor variables (step 2). Step 3 included the addition of HL, which explained an extra 23.6 percent of the variance. People with different levels of education and job status, self-efficacy, and perceived barriers show different reactions to the fear of the COVID-19 issue. Then, these factors are suggested to be applied to other communicable diseases and designing intervention programs in similar situations. This is where health policymakers and healthcare providers, etc., can play the most significant roles.

### Limitations

This study had some limitations. This research focused on the predictors of fear of COVID-19, which may have ignored the importance of other psychological factors. In addition, the study examined the determinants of fear of COVID-19 in a low-income country, which may not be generalizable to high-income countries. The variables measured with self-report questionnaires, the risk of highly subjective biases among participants may arise. Also, access to individuals was difficult during the COVID-19 pandemic.

## Conclusion

The present study aimed to investigate the predictors of fear of COVID-19 and HL importance among people. In total, the findings of this study highlight that self-efficacy and health literacy were determinants of fear of COVID-19. These findings can be helpful for health policymakers and healthcare providers in order to reduce the fear of COVID-19 and design HL-based programs in similar situations in the future.

## Data availability statement

The datasets presented in this study can be found in online repositories. The names of the repository/repositories and accession number(s) can be found in the article/supplementary material.

## Ethics statement

The studies involving humans were approved by Sarab Faculty of Medical Sciences. The studies were conducted in accordance with the local legislation and institutional requirements. The participants provided their written informed consent to participate in this study.

## Author contributions

SR: Methodology, Validation, Visualization, Writing – original draft, Writing – review & editing. KC: Data curation, Investigation, Methodology, Project administration, Validation, Writing – original draft, Writing – review & editing. SA-N: Formal analysis, Validation, Writing – original draft. TB: Formal analysis, Validation, Conceptualization, Methodology, Project administration, Supervision, Writing – original draft, Writing – review & editing.
